# Mortality Burden and Years of Life Lost Attributable to Air Pollution in Liguria, Italy: A Health Impact Assessment

**DOI:** 10.3390/jox16030114

**Published:** 2026-06-18

**Authors:** Sebastiano La Maestra, Francesco D’Agostini, Linda Ferrea

**Affiliations:** Department of Health Sciences (DiSSal), University of Genoa, 16132 Genoa, Italy; fda@unige.it (F.D.); linda.ferrea@edu.unige.it (L.F.)

**Keywords:** air pollution, health impact assessment, PM_2.5_, nitrogen dioxide, ozone, years of life lost

## Abstract

Air pollution is a major environmental determinant of premature mortality and population health burden. Liguria represents a vulnerable Mediterranean region due to intense urbanisation, port-related emissions, complex topography and an ageing population. This study quantified the mortality burden and Years of Life Lost (YLL) attributable to long-term exposure to PM_2.5_, NO_2_ and O_3_ in Liguria (Italy), and estimated the potentially avoidable burden under WHO guideline scenarios. A Health Impact Assessment (HIA) was conducted using ARPAL air quality data and ISTAT mortality data for the population aged ≥30 years during 2022–2024. Relative risks were derived from the European ELAPSE project and WHO meta-analyses. Attributable mortality was estimated using a log-linear Health Impact Function, while YLL were calculated using regional life tables and normalised per 100,000 inhabitants. PM_2.5_ was the main contributor to air pollution-related mortality, accounting for 1333 attributable deaths in 2022. Corresponding YLL ranged from 755 to 1012 per 100,000 inhabitants over the study period. NO_2_ showed a relevant but secondary contribution, while O_3_ effects were smaller and more uncertain. WHO guideline scenarios indicated a substantial potentially avoidable burden of deaths and YLL. These findings support targeted environmental and public health interventions in highly urbanised coastal regions.

## 1. Introduction

Air pollution is widely recognised as one of the most relevant environmental threats to public health worldwide. It contributes significantly to the burden of chronic non-communicable diseases and is also involved in the onset and exacerbation of acute conditions, particularly affecting the respiratory and cardiovascular systems. Globally, exposure to ambient air pollution is estimated to be responsible for approximately 4.2 million premature deaths each year, placing it among the leading risk factors for mortality alongside tobacco use and unhealthy diets [1,2].

Urban and suburban air pollution is characterised by a complex mixture of compounds, including particulate matter (PM), nitrogen oxides (NO_2_), sulphur dioxide (SO_2_), volatile organic compounds and other toxic substances. Within this mixture, three pollutants are commonly used as key indicators in epidemiological and environmental studies: fine particulate matter (PM_2.5_), nitrogen dioxide (NO_2_) and tropospheric ozone (O_3_). Although they differ in origin, chemical composition and atmospheric behaviour, all three have been consistently associated with increased mortality, mainly through biological pathways involving oxidative stress and systemic inflammation [3,4].

PM_2.5_ consists of particles with an aerodynamic diameter of 2.5 μm or less and represents one of the most harmful components of air pollution. Beyond particle size, PM_2.5_ toxicity is strongly influenced by its chemical composition and associated xenobiotic load. Fine particles may adsorb and transport polycyclic aromatic hydrocarbons (PAHs), transition metals, black carbon, quinones, aldehydes and other persistent organic pollutants generated by combustion sources, shipping emissions and industrial activities [5,6]. Several of these compounds exhibit redox activity and may activate xenobiotic-sensing pathways, including aryl hydrocarbon receptor (AhR)-dependent signalling and cytochrome P450 induction, leading to oxidative stress, mitochondrial dysfunction, DNA damage, endothelial injury and chronic inflammation [6,7]. These mechanisms are considered central in the pathogenesis of cardiovascular, respiratory and neoplastic diseases associated with long-term PM_2.5_ exposure [3,4,5,6,7,8,9]. A large body of epidemiological evidence has linked PM_2.5_ exposure to a wide range of health outcomes, including respiratory diseases, cardiovascular conditions and lung cancer [8,9]. Nitrogen dioxide (NO_2_) is primarily produced by combustion processes, especially those associated with road traffic, as well as by industrial and port activities. Exposure to NO_2_ has been associated with adverse respiratory effects, including increased asthma symptoms and higher respiratory mortality. In addition to its direct effects, NO_2_ plays an important role in atmospheric chemistry, serving as a precursor to ozone and secondary particulate matter, which, in turn, contribute to air pollution levels [10].

Ozone (O_3_), unlike PM_2.5_ and NO_2_, is not emitted directly but forms in the atmosphere through photochemical reactions involving nitrogen oxides and volatile organic compounds in the presence of sunlight. For this reason, its concentration shows a marked seasonal pattern, with higher levels during the warmer months. O_3_ exposure has been associated with increased mortality, particularly from respiratory causes, and its impact is expected to become more pronounced as climate change increases temperatures, which favour its formation [1,11,12]. Given the well-established health effects of air pollution, there has been increasing interest in methods that can translate epidemiological evidence into quantitative measures of population impact. In a preventive context, this translation is essential to support public health planning and policy decisions. Health Impact Assessment (HIA) is one of the most widely used approaches for this purpose, allowing the estimation of the number of health events attributable to specific environmental exposures [13,14]. The methodological basis of HIA is the Health Impact Function (HIF), which combines information on baseline health outcomes, exposure levels and concentration–response relationships derived from epidemiological studies. These relationships are typically expressed as relative risks (RRs) and describe how changes in pollutant concentrations are associated with variations in health outcomes [15]. An important aspect of this approach is the definition of a reference concentration, or counterfactual scenario, against which the health impact is evaluated [16]. While this value has traditionally been interpreted as a threshold below which no adverse effects occur, increasing evidence suggests that such a threshold may not exist, particularly for PM_2.5_, where health effects have been observed even at relatively low concentrations [17,18]. This has led to a revision of the World Health Organization (WHO) air quality guidelines, which now recommend substantially lower concentration levels [1,8].

Using these guideline values as a reference makes it possible not only to estimate the current burden of disease, but also to quantify the potential health benefits associated with improved air quality [19]. This is particularly relevant for regions with specific environmental and demographic characteristics [20].

**Figure 1:**
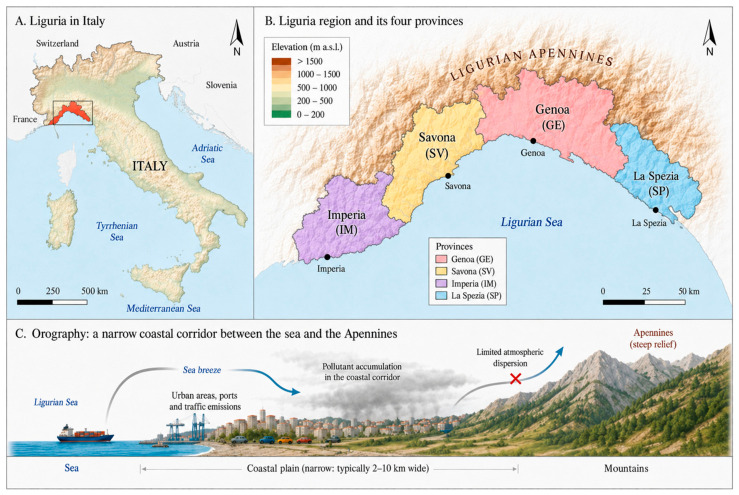
Study area characteristics of Liguria, Italy. (**A**) Location of Liguria in north-western Italy. (**B**) Liguria and its four provinces (Genoa, Savona, Imperia and La Spezia) with elevation gradients. (**C**) Schematic representation of the Ligurian coastal corridor, illustrating the proximity between urban and port areas and the Apennine mountain range, which may limit atmospheric dispersion and favour pollutant accumulation.

**Figure 2:**
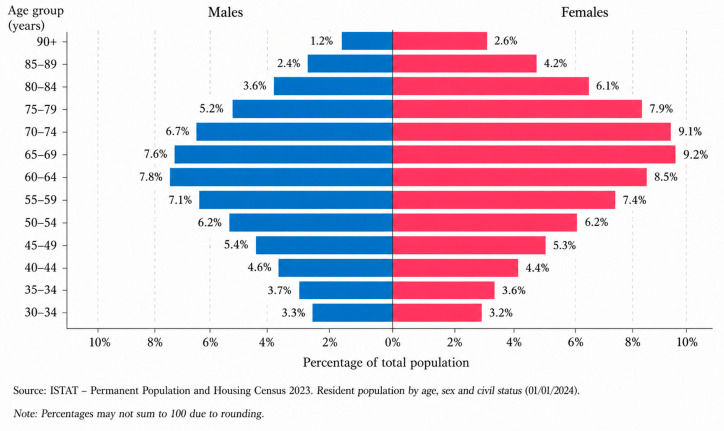
Population pyramid of residents aged 30 years or older in Liguria (2024). The figure highlights the marked ageing of the regional population, characterised by a high proportion of individuals aged 65 years and older and a predominance of women in the oldest age group.

Liguria represents an interesting case study in this context. The region is characterised by complex topography (Figure 1) and a relatively high proportion of elderly population (Figure 2), which may increase vulnerability to environmental exposures [21,22].

At the same time, the presence of urban areas, transport infrastructure and port-related activities contributes to spatial variability in air pollution levels [23]. Although several studies have investigated the health effects of air pollution at national or European levels, analyses at a sub-regional scale that combine recent exposure data with advanced indicators, such as Years of Life Lost (YLL), remain relatively limited [24]. Evidence at the provincial level is particularly scarce in Mediterranean regions characterised by the coexistence of intense urbanisation, port-related activities, complex topography and population ageing. YLL provides additional insight into the burden of disease by accounting not only for the number of deaths but also for the age at which they occur [25,26].

In this context, the present study aims to estimate the mortality burden attributable to long-term exposure to PM_2.5_, NO_2_ and O_3_ in Liguria, using a HIA based on recent epidemiological evidence and provincial-level data. In addition to attributable deaths, the study quantifies YLL and evaluates the potential reduction in health burden under a scenario in which pollutant concentrations are reduced to the levels recommended by the WHO.

## 2. Materials and Methods

### 2.1. Study Design

HIA was conducted to estimate the mortality burden attributable to long-term exposure to air pollution in the Liguria region. Liguria is a coastal region in north-western Italy, facing the Ligurian Sea, characterised by a densely populated, highly urbanised coastal area bounded by the sea and the Apennine Mountains. The analysis covered the period 2022–2024 and considered all provinces, including Genoa, Savona, Imperia, and La Spezia. The geographical location of Liguria, its provincial boundaries, and the main topographic characteristics that potentially influence pollutant dispersion are shown in Figure 1. The age structure of the Ligurian population is shown in Figure 2. Consistent with official census data, the region is characterised by a markedly aged population, with an age profile substantially older than the national average.

### 2.2. Exposure Assessment

Annual mean concentrations of fine particulate matter (PM_2.5_) and NO_2_, and warm-season (April–September) mean concentrations of O_3_, were obtained from published data of the Ligurian Regional Environmental Protection Agency (ARPAL) [27]. O_3_ exposure was defined as the April–September average concentration available from ARPAL monitoring data. For each pollutant, province-level average concentrations were calculated for each year of the study period.

All available ARPAL monitoring stations within each province were included in the analysis, and provincial concentrations were obtained as arithmetic means of all valid measurements. The number of monitoring stations contributing to the provincial averages was 8–9 PM_2.5_, 19–20 NO_2_ and 5–6 O_3_ stations in Genoa; 12 PM_2.5_, 14 NO_2_ and 3 O_3_ stations in Savona; 1 PM_2.5_, 4 NO_2_ and 2–3 O_3_ stations in Imperia; and 4 PM_2.5_, 13–14 NO_2_ and 3 O_3_ stations in La Spezia. For O_3_, only observations collected between April and September were considered, consistent with the warm-season exposure metric adopted in the study. Missing or non-valid observations were excluded from the calculations, and no imputation procedures were applied. No direct chemical speciation data for PM_2.5_-bound xenobiotics were available at the provincial scale for the study period; therefore, PM_2.5_ mass concentration was used as the exposure indicator. Health impacts were estimated only for exposure levels exceeding the concentrations suggested by the WHO guidelines: 5 μg/m^3^ for PM_2.5_, 10 μg/m^3^ for NO_2_, and 60 μg/m^3^ for O_3_ [1].

### 2.3. Mortality Data

All-cause mortality data for individuals aged ≥30 years were obtained from the Italian National Institute of Statistics (ISTAT), stratified by province and year. Since cause-specific mortality data were not available at the provincial level, a 4% correction factor was applied to approximate natural mortality by reducing the contribution of deaths from external causes. This value was selected based on Italian mortality statistics, where deaths due to external causes (ICD-10 V01–Y89) generally account for approximately 4–6% of total mortality, and was considered a conservative estimate for Liguria given its particularly aged population structure [28].

### 2.4. Concentration–Response Functions

Relative risks for long-term exposure to PM_2.5_ and NO_2_ were derived from the European ELAPSE study [18], while estimates for O_3_ were based on WHO systematic reviews [29]. The RR values used in the analysis are reported in Table 1.

Assuming a log-linear relationship between pollutant concentration and mortality, the exposure–response coefficient (β) was calculated as:

$$\beta =\frac{ln(RR)}{10}$$

### 2.5. Health Impact Function

Attributable deaths (AC) were estimated using a standard Health Impact Function:

$$AC={(D}_{\geq 30}\times 0.96)\times (1-e^{-\beta (E-t)})$$

where:

- $D_{\geq 30}$ is the number of deaths among individuals aged ≥30 years;
- 0.96 accounts for the exclusion of external causes (−4%);
- E is the observed pollutant concentration;
- t is the reference (counterfactual) concentration based on WHO guidelines;
- β is the exposure–response coefficient.

Only positive differences between E and t were considered.

### 2.6. Years of Life Lost (YLL)

YLL were calculated to quantify the burden of premature mortality. Attributable deaths were allocated across age groups proportionally to the observed age-specific mortality distribution available from ISTAT mortality statistics (0–49, 50–64, 65–79 and ≥80 years). Age-specific attributable deaths were then multiplied by the corresponding remaining life expectancy derived from regional life tables for Liguria [30].

$$YLL=\sum_{a}AC_{a}\times e_{x,a}$$

where $AC_{a}$ represents the attributable deaths allocated to age group a and $e_{a}$ the corresponding remaining life expectancy. YLL were subsequently normalised per 100,000 inhabitants aged ≥30 years.

### 2.7. Counterfactual Scenario and Statistical Considerations

To estimate the potential health benefits of improved air quality, a counterfactual scenario was defined in which pollutant concentrations were reduced to WHO guideline values. Avoidable deaths and YLL correspond to the attributable burden above these thresholds.

All analyses were conducted under the assumption of single-pollutant models. Due to the correlation between pollutants, results were not summed across pollutants to avoid double-counting.

Uncertainty was estimated using the 95% confidence intervals of the relative risks, propagated through the health impact function.

### 2.8. AI-Assisted Image Generation

Artificial intelligence (AI)-assisted image-generation tools were used exclusively to prepare the graphical abstract and selected illustrative figures. All generated images were subsequently reviewed, edited and validated by the authors to ensure consistency with the study design and scientific content.

## 3. Results

### 3.1. Air Pollution Concentrations

Air pollutant concentrations varied across provinces and over the study period, reflecting both spatial heterogeneity and temporal trends (Table 2).

PM_2.5_ and NO_2_ concentrations decreased consistently between 2022 and 2024 across all provinces, whereas O_3_ showed greater interannual variability.

PM_2.5_ concentrations ranged from 7.04 to 11.17 μg/m^3^, with the highest values observed in Genoa in 2022 and the lowest in Imperia in 2024. All recorded PM_2.5_ levels exceeded the WHO guideline value of 5 μg/m^3^. NO_2_ concentrations ranged from 7.76 to 25.25 μg/m^3^, with higher levels in urban areas, particularly in Genoa. Concentrations exceeded the WHO guideline value of 10 μg/m^3^ in most provinces and years, except in Imperia in 2024.

O_3_ concentrations, calculated as warm-season averages, were consistently above the WHO reference value of 60 μg/m^3^, ranging from 70.33 to 93.12 μg/m^3^. The highest O_3_ levels were observed in 2023, suggesting that meteorological conditions strongly influence its formation.

Overall, the data indicate a general improvement in PM_2.5_ and NO_2_ levels over time, while O_3_ trends appear more variable and less directly linked to emission reductions.

In particular, the largest reduction was observed for NO_2_ concentrations in Genoa, decreasing from 25.25 μg/m^3^ in 2022 to 21.18 μg/m^3^ in 2024.

### 3.2. Attributable Mortality

A substantial number of deaths were attributable to long-term exposure to air pollution across all provinces and years, as reported in Table 3.

PM_2.5_ was consistently the dominant contributor to mortality burden, accounting for 1333 attributable deaths in Liguria in 2022, decreasing to 991 in 2023 and 939 in 2024.

NO_2_ also contributed significantly to mortality, with 1012 attributable deaths in Liguria in 2022, followed by 708 in 2023 and 616 in 2024. The magnitude of the NO_2_-related estimates was particularly relevant in Genoa, where concentrations and population density were highest.

In contrast, the impact of O_3_ was smaller, with 488 attributable deaths in Liguria in 2022, rising to 560 in 2023, and then decreasing to 391 in 2024. However, the wide confidence intervals for O_3_, often including the null value, indicate greater uncertainty in these estimates than for PM_2.5_ and NO_2_.

At the provincial level, Genoa consistently accounted for the largest proportion of attributable deaths for both PM_2.5_ and NO_2_, reflecting both higher exposure levels and demographic characteristics. Savona and La Spezia showed intermediate values, while Imperia presented lower contributions, particularly for NO_2_, with negligible estimates in 2024.

These findings highlight a clear spatial gradient, with higher mortality burden observed in more urbanised and densely populated areas. Across all years, PM_2.5_ accounted for the highest number of attributable deaths compared to NO_2_ and O_3_ in all provinces.

### 3.3. Years of Life Lost (YLL)

The burden of disease expressed as YLL showed patterns consistent with those observed for attributable mortality (Table 4).

When normalised per 100,000 inhabitants aged ≥30 years, PM_2.5_-related YLL in Liguria ranged from 1012 in 2022 to 755 in 2024, indicating a substantial impact on population health.

NO_2_-related YLL ranged from 768 to 491 per 100,000 inhabitants, confirming its role as a relevant, although secondary, contributor to the overall burden. O_3_-related YLL were lower, ranging from 371 to 314 per 100,000 inhabitants, with a more variable trend over time.

The decline in YLL associated with PM_2.5_ and NO_2_ between 2022 and 2024 reflects the reduction in exposure levels observed in the same period. In contrast, the variability of O_3_-related YLL appears to be driven more by interannual changes in environmental conditions than by consistent trends in emissions. The highest YLL values were consistently associated with PM_2.5_ exposure, particularly in 2022.

### 3.4. Counterfactual Scenario and Avoidable Burden

The attributable burden estimated in this study can be interpreted as the number of deaths and YLL potentially avoidable under a counterfactual scenario in which pollutant concentrations are reduced to WHO guideline values. The avoidable burden was highest for PM_2.5_, followed by NO_2_, while estimates for O_3_ were smaller and more uncertain. In terms of YLL, the largest potential reduction was observed for PM_2.5_, highlighting the substantial public health benefits that could be achieved through further improvements in air quality.

## 4. Discussion

This study provides a detailed assessment of the mortality burden attributable to long-term exposure to air pollution in Liguria, based on recent exposure data and updated concentration–response functions. The results consistently indicate that PM_2.5_ represents the main contributor to both attributable mortality and YLL, followed by NO_2_, while O_3_ shows a smaller and more uncertain impact.

The magnitude of the PM_2.5_-related burden observed in this study is consistent with previous health impact assessments conducted at the European level, including those reported by the European Environment Agency, which identify fine particulate matter as the dominant contributor to air pollution-related mortality. In Liguria, PM_2.5_ concentrations were consistently above the WHO guideline value of 5 μg/m^3^, as reflected in the relatively high number of attributable deaths and YLL estimated across all provinces. When expressed per 100,000 inhabitants aged ≥30 years, the burden associated with PM_2.5_ exceeded 1000 YLL in 2022, confirming the substantial public health impact of this pollutant even in regions with moderate concentrations.

The decreasing trend observed between 2022 and 2024 for both attributable mortality and YLL associated with PM_2.5_ suggests that reductions in exposure levels are associated with measurable improvements in population health. Since attributable mortality depends on both exposure levels and baseline mortality, temporal variations in mortality patterns may have contributed to the observed decrease in burden. The inclusion of YLL provides additional insight beyond mortality counts alone, as it accounts for age at death and therefore better captures the overall loss of population health.

This finding is consistent with previous studies showing that even relatively small decreases in PM_2.5_ concentrations can lead to significant reductions in mortality burden. The burden estimated in Liguria should also be interpreted in the context of previous city-level Health Impact Assessments conducted in Europe. Khomenko et al. analysed 969 European cities and 47 larger cities. They identified substantial geographical variability in the health burden attributable to air pollution, with the highest PM_2.5_-related burden observed in northern Italy, Poland and the Czech Republic, and the highest NO_2_-related burden in large urban areas of western and southern Europe, including Madrid, Paris, Milan and Barcelona [19]. In that study, PM_2.5_-related Years of Life Lost (YLL) exceeded 1700 per 100,000 inhabitants in some highly polluted cities of the Po Valley, such as Brescia and Bergamo, whereas substantially lower values were reported in northern European cities [19]. The PM_2.5_-related YLL estimated in Liguria (755–1012 per 100,000 inhabitants aged ≥30 years) are therefore lower than those reported for the most heavily polluted urban areas of northern Italy, but remain substantial despite comparatively lower pollutant concentrations. This finding suggests that factors other than exposure levels alone may contribute to the observed burden. In particular, Liguria is characterised by one of the oldest population structures in Europe, elevated baseline mortality, intense urbanisation and the concentration of transport and port-related activities along a narrow coastal corridor. These characteristics may increase population vulnerability and help explain why a considerable burden persists even in the absence of the extreme pollution levels reported for some industrialised areas of Europe. For NO_2_, the predominance of the burden in Genoa is consistent with patterns observed in other large Mediterranean urban areas where traffic density and maritime activities contribute substantially to the urban pollution mixture.

From a toxicological perspective, PM_2.5_ should be considered not only as a mass-based exposure indicator but also as a carrier of biologically active xenobiotics. Fine particles are known to transport a wide range of combustion-derived compounds, including polycyclic aromatic hydrocarbons, transition metals and oxidised organic species. Although chemical speciation was not available in the present study, the observed burden is consistent with the established evidence linking PM_2.5_-associated xenobiotic mixtures to adverse cardiovascular and respiratory outcomes. Several mechanisms have been proposed to explain the health effects associated with PM_2.5_ exposure, including oxidative stress, systemic inflammation, endothelial dysfunction and altered cellular responses to xenobiotic compounds. While the present analysis does not allow attribution of the observed burden to specific chemical constituents, these mechanisms provide biological plausibility for the associations reported in epidemiological studies. Future investigations integrating PM_2.5_ mass concentrations with chemical speciation and source-apportionment approaches may help better characterise the contributions of specific emission sources in Liguria. NO_2_ also contributed to the overall burden, particularly in Genoa, where higher concentrations and population density are observed.

This pattern reflects the region’s specific emission profile, in which traffic and port-related activities are major sources of nitrogen oxides. The relevance of NO_2_ in this context is further supported by its spatial distribution, which closely follows urbanisation patterns. However, as NO_2_ is strongly correlated with PM_2.5_, its contribution should be interpreted with caution. The use of single-pollutant models may lead to partial overlap in estimated effects, and NO_2_ may serve as an indicator of the broader urban pollution mixture rather than a fully independent causal factor.

In contrast, the contribution of O_3_ appears more limited and is characterised by greater uncertainty. Although O_3_ concentrations were consistently above the WHO reference value, the associated estimates had wider confidence intervals, often including the null value, indicating a less robust association than for PM_2.5_ and NO_2_. The variability observed across years, with higher values in 2023, likely reflects the strong influence of meteorological conditions and photochemical processes on ozone formation. This suggests that, unlike PM_2.5_ and NO_2_, O_3_ trends are less directly linked to emission reductions and more dependent on environmental factors.

The spatial distribution of the burden reflects the unique geographical and socio-demographic characteristics of Liguria. The region is characterised by a highly anthropised coastal strip, where most of the population and economic activities are concentrated. This configuration results in higher exposure levels in urban areas, particularly in Genoa, which consistently shows the highest attributable mortality and YLL values. In contrast, provinces such as Imperia, with lower population density and reduced traffic intensity, show lower NO_2_ contributions and a relatively smaller overall burden. The higher burden observed in Genoa is likely explained by the combined effect of higher pollutant concentrations, greater population density and an older population structure, factors that may increase both exposure and susceptibility to air pollution-related health effects. Furthermore, the region’s geographical and topographical characteristics (Figure 1), including the narrow coastal corridor bounded by the Ligurian Sea and the Apennine mountain range, may contribute to limited atmospheric dispersion and favour the accumulation of pollutants in densely populated urban areas. These features may partly explain the spatial distribution of the estimated burden across the region.

Liguria also presents one of the highest ageing indices in Europe, with a large proportion of the population aged 65 years and over. This demographic structure likely amplifies the observed health impacts, as older individuals are more vulnerable to air pollution’s effects, particularly on cardiovascular and respiratory outcomes. This demographic profile is also reflected in the population pyramid shown in Figure 2, which highlights the substantial proportion of older residents and the predominance of women in the oldest age classes. The combined effect of population ageing and spatial concentration of emissions may therefore contribute to the magnitude of the burden observed in the region. Consequently, the relatively high burden observed in Liguria is likely driven by the interaction between population ageing, baseline mortality and persistent exposure to air pollutants, rather than by exposure levels alone.

The counterfactual analysis indicates that achieving WHO guideline values would result in a substantial reduction in both attributable deaths and YLL. This finding highlights the significant potential for public health gains associated with further improvements in air quality. In particular, reductions in PM_2.5_ concentrations appear to offer the greatest benefit, consistent with its dominant contribution to the overall burden. These results support the need for targeted interventions to reduce emissions from key sources, including road traffic, port activities, and domestic heating systems.

Some limitations of this study should be acknowledged. First, using province-level average concentrations may underestimate intra-urban variability and fail to capture high-exposure hotspots, such as areas near major roads or industrial facilities. Second, the analysis was based on single-pollutant models, and results should not be summed across pollutants to avoid double-counting. Third, although WHO guideline values were used as reference concentrations, epidemiological evidence suggests that no clear threshold exists, particularly for PM_2.5_, and health effects may occur even at lower concentrations. Finally, uncertainty in the estimates is primarily related to the concentration–response functions and exposure assessment, although these are consistent with current HIA methodologies. Additional uncertainty affects the YLL estimates, which depend on the allocation of attributable deaths across age groups and on the use of regional life tables to estimate remaining life expectancy. Although these approaches are commonly adopted in health impact assessments, they may not fully capture individual variations in survival and mortality patterns. This aspect is particularly relevant in Liguria, where the marked ageing of the population may influence the distribution of both mortality and years of life lost. Despite these limitations, the study provides robust, locally relevant evidence on the health impacts of air pollution in Liguria. By combining attributable mortality with YLL, the analysis provides a more comprehensive assessment of population health loss and underscores the importance of continued efforts to reduce air pollution in the region.

## 5. Conclusions

This study provides locally relevant evidence on the health burden attributable to long-term exposure to air pollution in Liguria. PM_2.5_ was the main contributor to attributable mortality and YLL, while NO_2_ showed a relevant contribution in urban areas, and O_3_ estimates were more uncertain. Achieving the guideline levels would prevent a substantial burden of deaths and years of life lost. These findings support targeted air quality policies, particularly in densely populated, highly anthropised coastal areas. The results also highlight the importance of considering local demographic and geographical characteristics when evaluating the health impacts of air pollution. In regions such as Liguria, characterised by population ageing, intense urbanisation and limited atmospheric dispersion, targeted emission-reduction strategies may yield substantial public health benefits and help reduce the burden of environmentally related disease. Particular attention should be paid to major urban and port areas such as Genoa, where the coexistence of dense population, transport infrastructure and maritime activities may substantially contribute to long-term exposure and associated health impacts.

## Figures and Tables

**Table 1:** Summary of concentration–response parameters.

Pollutant	Exposure Metric	RR (per 10 μg/m^3^)	95% CI	β	Counterfactual (μg/m^3^)
PM_2.5_	Annual mean	1.118	1.060–1.179	0.01116	5
NO_2_	Annual mean	1.045	1.026–1.065	0.00440	10
O_3_	Warm-season mean	1.010	1.000–1.020	0.000995	60

**Table 2:** Annual mean PM_2.5_ and NO_2_ concentrations and warm-season O_3_ concentrations (μg/m^3^) by province and year.

Province	PM_2.5_ μg/m^3^ 2022	PM_2.5_ μg/m^3^ 2023	PM_2.5_ μg/m^3^ 2024	NO_2_ μg/m^3^ 2022	NO_2_ μg/m^3^ 2023	NO_2_ μg/m^3^ 2024	O_3_ μg/m^3^ 2022 *	O_3_ μg/m^3^ 2023 *	O_3_ μg/m^3^ 2024 *
Genoa	11.17	9.42	9.72	25.25	22.75	21.18	84.72	93.12	85.60
Savona	10.14	9.04	9.03	16.33	13.24	12.08	76.99	82.42	70.33
Imperia	8.16	9.57	7.04	11.10	10.19	7.76	85.45	87.67	78.00
La Spezia	9.92	9.67	10.17	15.84	13.66	14.80	71.80	72.86	70.79

* O_3_ concentrations refer to warm-season (April–September) averages.

**Table 3:** Number of attributable deaths (*n*) by pollutant, province and year (95% CI).

**Province**	**PM_2.5_** **2022**	**PM_2.5_** **2023**	**PM_2.5_** **2024**
Genoa	823 (437–1196)	535 (283–781)	560 (296–816)
Savona	230 (122–335)	168 (89–245)	161 (85–236)
Imperia	113 (59–165)	145 (77–212)	63 (33–92)
La Spezia	167 (88–243)	142 (75–207)	155 (82–226)
Liguria	1333 (707–1940)	991 (524–1446)	939 (496–1370)
**Province**	**NO_2_** **2022**	**NO_2_** **2023**	**NO_2_** **2024**
Genoa	804 (475–1134)	606 (358–857)	524 (309–742)
Savona	114 (67–162)	54 (32–77)	33 (20–48)
Imperia	16 (9–22)	2 (1–3)	0 (0-0)
La Spezia	79 (46–113)	45 (26–64)	58 (34–83)
Liguria	1012 (597–1430)	708 (417–1001)	616 (362–873)
**Province**	**O_3_** **2022**	**O_3_** **2023**	**O_3_** **2024**
Genoa	301 (0–591)	360 (0–705)	275 (0–540)
Savona	69 (0–137)	84 (0–166)	38 (0–74)
Imperia	81 (0–160)	80 (0–156)	49 (0–97)
La Spezia	36 (0–72)	36 (0–70)	30 (0–59)
Liguria	488 (0–960)	560 (0–1098)	391 (0–770)

**Table 4:** Years of Life Lost (YLL per 100,000 inhabitants aged ≥30 years) by pollutant and year in Liguria.

Pollutant	2022	2023	2024
PM_2.5_	1012	789	755
NO_2_	768	561	491
O_3_	371	446	314

## Data Availability

The data presented in this study are available from publicly accessible repositories. Air quality data were obtained from the ARPAL Air Quality Dashboard: Public Monitoring Data (https://aria-dashboard.regione.liguria.it/reports/powerbi/Deliverable/Qualita_Aria/Pubblico/Ambiente_dati_publicati_dl; accessed on 7 May 2026). Mortality data were derived from the Italian National Institute of Statistics (ISTAT) Mortality and Causes of Death Database (https://www.istat.it/en/news/mortality-data/; accessed on 7 May 2026). Life expectancy data used for the Years of Life Lost calculations were obtained from the ISTAT Life Tables of the Resident Population (https://demo.istat.it/app/?i=TVM&l=en; accessed on 1 April 2026).
